# Influence of Emergency Situations on Maternal and Infant Nutrition: Evidence and Policy Implications from Hurricane John in Guerrero, Mexico

**DOI:** 10.3390/ijerph22111615

**Published:** 2025-10-23

**Authors:** Edith Kim-Herrera, Ana Lilia Lozada-Tequeanes, Dinorah González-Castell, Edgar Arturo Chávez-Muñoz, Rocío Alvarado-Casas, Susana Rafalli-Arismendi, Matthias Sachse-Aguilera, Cecilia De Bustos, Anabelle Bonvecchio-Arenas

**Affiliations:** 1 Nutrition and Health Research Center (CINyS), National Institute of Public Health of Mexico (NIPH), Avenida Universidad 655, Santa María Ahuacatitlán, Cuernavaca 62100, Mexico; edith_k10@hotmail.com (E.K.-H.); cinys20@insp.mx (A.L.L.-T.); lgonzalez@insp.mx (D.G.-C.); nutpedia.chavezm@gmail.com (E.A.C.-M.); alvacaro0204@gmail.com (R.A.-C.); 2 Independent Consultant, Avenida Teherán. Urb. Montalbán, Cáritas, Caracas 2042, Venezuela; susana.raffalli@gmail.com; 3 United Nations International Children’s Emergency Fund (UNICEF) of Mexico, Avenida Paseo de la Reforma 645, Lomas de Chapultepec, Miguel Hidalgo, Ciudad de México 11000, Mexico; msachse@unicef.org (M.S.-A.); cdebustos@unicef.org (C.D.B.)

**Keywords:** infant feeding, breastfeeding, complementary feeding, pregnancy, nutritional status, emergency, disaster, Mexico

## Abstract

In emergencies, the maternal and child populations face increased risk of morbidity and mortality, often exacerbated by malnutrition. Breastfeeding, adequate complementary feeding, and appropriate prenatal care can mitigate these risks. This descriptive cross-sectional study compared data collected before and after Hurricane John related to maternal, infant and young child feeding (IYCF) practices and the nutritional status of pregnant women and children under two years of age. Data were collected in December 2024 from the two provinces most affected in Guerrero, Mexico. Surveys were completed for 239 children through caregivers and 76 pregnant women, alongside anthropometric assessments. After the disaster, findings showed a significant decline in breastfeeding among 0–6-month-olds (88.7% to 71.1%) and an increase in dietary diversity in complementary feeding (3.6 ± 2.1 vs. 4.5 ± 1.5 food groups). Malnutrition, based on weight-for-length z-scores, was observed in 4.8% of children aged 0–6 months and 2.6% of those aged 6–24 months. According to mid-upper arm circumference, 8.4% of children aged 0–6 months presented malnutrition. Among pregnant women, based on body mass index, 41.5% had excessive pre-pregnancy weight, while 12.3% were underweight. These findings underscore the urgent need to integrate maternal and child nutrition into emergency preparedness and response strategies to protect vulnerable populations in Mexico.

## 1. Introduction

In emergency contexts, infants and young children face an increased risk of morbidity and mortality, further exacerbated by malnutrition and the interruption of breastfeeding [1,2]. Appropriate infant and young child feeding (IYCF) practices are therefore essential to safeguard health, particularly during emergencies [3]. Likewise, pregnant women constitute a particularly vulnerable group in such contexts due to the specific physiological and nutritional demands of pregnancy. According to the World Health Organization (WHO), pregnant women should receive timely prenatal care and adequate micronutrient supplementation to prevent complications for both the mother and the fetus under all circumstances, including emergencies [4].

Evidence suggests that disasters from natural hazards and human-made disasters negatively affect IYCF practices and the nutritional status of both children and pregnant women living in affected areas [5,6]. These impacts have been particularly documented in Asian [7] and African countries [8], while evidence from low- and middle-income countries in Latin America [9,10] remains scarce.

Some studies focus on the difficulties faced by disaster responders and mothers for optimal infant feeding during disasters in middle and high-income countries have highlighted a major challenge faced by organizations implementing infant feeding in emergencies [11]: frequent violations of the International Code of Marketing of Breastmilk Substitutes (hereinafter referred to as the Code) by other aid organizations and government agencies, including the acceptance of donated commercial infant formula (CIF) and its untargeted distribution. Furthermore, evidence indicates that most disaster response efforts lack adequate familiarity with established infant feeding in emergencies protocols [12,13].

As documented by the United Nations Children’s Fund (UNICEF) following a natural disaster in Indonesia, despite high rates of breastfeeding initiation, 70% of infants under six months of age received donated CIF [14]. Similarly, the Centers for Disease Control and Prevention (CDC) investigated post-flood deaths of more than 500 children in Botswana in 2005–2006 and found that nearly all infants who died had been fed with CIF. The risk of hospitalization among these infants was estimated to be 50 times higher than among breastfed infants [15].

Cultural norms and traditions shape breastfeeding practices in routine settings and become particularly influential in emergency contexts [16]. Additionally, complementary feeding in emergencies is often disrupted by damaged markets, water infrastructure, and health services, which limit access to nutrient-rich foods and safe food preparation. Families frequently struggle to meet children’s nutritional needs due to food shortages, reduced purchasing power, lack of cooking fuel, and poor sanitation [17,18]. These cha-llenges are compounded by exposure to inadequate water, sanitation, and hygiene (WASH) conditions, which increase the incidence of diarrhea [11], a condition responsible for up to 90% of child deaths in emergency situations [19].

In Mexico, as in many regions across the Americas and worldwide, the frequency of high-magnitude disasters, such as hurricanes and earthquakes, has been increasing, resulting in significant socioeconomic impacts and substantial losses [20]. On October 19, 2023, the population along the Guerrero coast in southeastern Mexico was belatedly alerted to the approach of a major hurricane, named Otis. This event caused severe damage among vulnerable populations in Guerrero [21]. Later, in September 2024, Hurricane John made landfall in the state as a Category 3 storm, with sustained winds of up to 195 km per hour. The hurricane caused extensive destruction, including catastrophic flooding and mudslides, particularly affecting Acapulco and surrounding areas. Approximately 270,000 people were impacted, and 23 fatalities were reported [22], exacerbating existing social and health disparities in the region. Although the most severe impact was concentrated along the coast, the emergency triggered large-scale response efforts, including the distribution of over 100,000 food packages and the deployment of health mobile units providing services to the population throughout the state.

In Mexico, evidence on the effects of natural disasters on maternal and infant nutrition remains limited, particularly studies comparing IYCF practices before and after an emergency. In response to the most recent tropical cyclones that struck Guerrero, there is a pressing need to strengthen state-level preparedness and response capacities. This includes the development of a plan or protocol that defines clear objectives and interventions to protect and promote maternal and child nutrition in such contexts. Furthermore, the establishment of a Maternal and Child Nutrition Technical Working Group for emergencies has been proposed, bringing together key institutions involved in nutrition and risk management to operationalize this plan, coordinate actions, and ensure its implementation across all phases of future emergencies.

The evidence underscore the need for local authorities to incorporate lessons learned from previous experiences and to align emergency responses with international guidelines and standards, including the Operational Guidance on Infant and Young Child Feeding in Emergencies (OG-IFE) [3] and the humanitarian responses of Sphere Standards [23], to ensure effective and coordinated actions. In this context, the objective of this study was to document and examine the nutritional status and feeding practices of pregnant women and children under two years of age (0–6 months and 6–24 months) after Hurricane John.

## 2. Materials and Methods

### 2.1. Design and Participants

This descriptive cross-sectional study compares conditions before and after Hurricane John to assess maternal, IYCF practices and the nutritional status of pregnant women and children under two years of age. Data were collected through a single survey conducted two months after the hurricane, as part of a situational diagnosis following the hurricane. The survey assessed current pregnancy and IYCF practices at the time of data collection (after-hurricane) and included retrospective questions regarding these practices one week prior to the hurricane (before-hurricane). The study was conducted in two pro-vinces of Guerrero, Mexico, affected by a major natural disaster.

**Figure 1:**
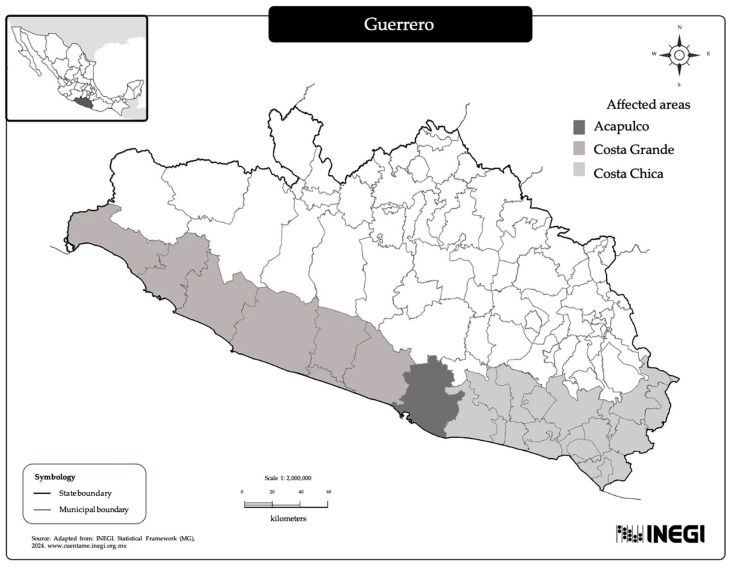
Map of Coast, Guerrero, Southern Mexico and affected areas.

The study population was drawn from 20 primary healthcare units in two provinces (Acapulco and Costa Grande) within the Guerrero State Health Ministry (Secretaría de Salud de Guerrero, SSGro by its acronym in Spanish) (Figure 1) [24]. These healthcare units were selected based on SSGro criteria, which prioritized those areas that were most affected by Hurricane John and had the largest maternal and child user populations. Only zones that were considered safe for fieldwork were selected. The target population using the selected healthcare units was identified and encouraged to participate during the information gathering, through outreaches by health workers. The inclusion criteria for participants were as follows: (a) being a resident of the area affected by Hurricane John; (b) having a child or being the primary caregiver of a child within the target age group (0–24 months) at the time of the emergency; (c) being pregnant or breastfeeding during the emergency; (d) being a user of one of the selected healthcare units; and (e) not presenting any mental impairment or physical disability that would prevent participation in anthropometric measurements or survey responses. The study population was divided into three groups: (1) pregnant women; (2) children aged 0 to 5.9 months and their mothers or caregivers; and (3) children aged 6 to 24 months and their mothers or caregivers. A statistical sample size calculation was not conducted. Instead, data collection was carried out in healthcare units with the highest number of registered maternal and child users to maximize participant recruitment during the study period. Participants who remained at home after the emergency represented the most vulnerable population, as their limited mobility reflected lower economic and social resources [25]. This characteristic may suggest that the sample studied include the most affected population.

### 2.2. Data Collection

Data were collected during December 2024, a little over two months after the Hurricane John struck Guerrero, through a single survey directed to pregnant woman or principal caregiver of children under 24 months of age through a single survey using previously validated questionnaires, which were adapted to obtain information before and after the emergency [26,27,28,29]. To ensure the validity and reliability of these instruments, field staff received standardized training prior to data collection, and the questionnaires were pilot-tested in a population with characteristics similar to those of the study participants to assess comprehension and feasibility.

Sociodemographic information was asked: age, schooling, family composition, socioeconomic level of the household, and marital status. Likewise, the instrument documented the before and after the emergency regarding health care (nutritional or breastfeeding guidance for health services), food support (donations, pantries), access to water, and maternal and IYCF practices and micronutrients supplementation reception, as well as some indicators of food poverty, among other sociodemographic and contextual risk factors (i.e., employment, livelihoods).

Information on IYCF practices was collected by asking whether she was “currently breastfeeding” (before and after the emergency) and whether she was “exclusively breastfeeding” at the time of data collection (after emergency). Additionally, mothers were asked whether their child was “consuming any other type of liquid or food group” (CIF, plain water, non-human milk, non- and sugar beverages, cereals, legumes, fruits, vegetables, meats, sausages, eggs, dairy, and snacks group (chips, sweets, cookies), both before and after the emergency. To calculate the exclusive breastfeeding indicator [29], we corroborated no consumption of any other type of liquid or food group different from breast milk. To estimate the indicator for any type of breastfeeding, we analyzed how mothers fed their babies before and after the emergency, complemented by the variable measuring the consumption of any other liquids or food groups.

For children aged 6–24 months, dietary diversity was assessed, for consistency, using the same food groups of National Health and Nutrition Survey (ENSANUT by its acronym in Spanish) consumed before and after the emergency. Six food groups were considered as follows: breast milk, cereals and legumes, fruits and vegetables, meat (red meat and other meats), eggs and dairy products. Children who consumed four or more of these food groups were classified as having adequate dietary diversity [28].

Anthropometric measurements were taken of children under 2 years of age, using standardized techniques for weight, height/length, and mid-upper arm circumference (MUAC). Previously calibrated instruments were used for this purpose; that is, electronic scales with a precision of 50 g and height/length, using a stadiometer with a precision of 1 mm (SECA, Hamburg, Germany). In addition, standardized tapes were used to measure MUAC (UNICEF, New York, NY, USA) [30]. In this sense, all personnel in charge of collecting quantitative information were trained and standardized in both anthropometric measurement techniques and the administration of data collection instruments. This training was conducted through a practical workshop held at the National Institute of Public Health (NIPH) of Mexico, which included hands-on exercises in data collection and anthropometric assessment. Field work was supervised by the project coordinator to ensure quality and consistency. Anthropometric measurements were taken only once, at the time of data collection (after hurricane).

Field work staff who were nutritionists applied the surveys using mobile tablets and recorded responses electronically through a custom-designed data entry form in RedCap (Research Electronic Data Capture; Vanderbilt University, Nashville, TN, USA) [31]. These digital forms were pre-validated to minimize data entry errors. In addition, data was reviewed at the end of each workday to ensure that all information had been correctly entered and saved. The databases were backed up online daily until data collection was completed.

### 2.3. Data Analysis

A descriptive analysis was performed in which sociodemographic, health, nutrition, access to water, and maternal and IYCF practices were considered. The normality of continuous variables was assessed using the Shapiro–Wilk test. For categorical and quantitative variables, percentages were estimated, while for continuous variables, means and standard deviation (SD) were estimated. Sociodemographic variables were categorized as follows: women’s age was grouped into four categories: 16–24 years, 25–29 years, 30–39 years, and 40 years and older. Marital status was categorized into five main groups: “Free union” (individuals living under the same roof or in the same household without being formally married), married, single, separated/divorced, and widowed. Educational level was categorized based on the highest level completed as: none, primary, secondary, high school, technical or commercial studies and a bachelor’s degree. The classification of socioeconomic status (SES) [27] was obtained by estimating the level of satisfaction of the most important needs of the household, considering six characteristics and household assets. SES was classified according to the Mexican Association of Market Research Agencies (AMAI by its acronym in Spanish). The six variables are: Human Capital, Practical Infrastructure, Connectivity and Entertainment, Healthcare Infrastructure, Planning and Future and Basic Infrastructure and Space. Finally, SES was grouped into four categories: high, medium, low, and very low SES.

Regarding anthropometric information, all children-related available data was analyzed according to age in two groups: infants of 0–6 months and children 6–24 months of age. Nutritional status indices were calculated through the WHO Anthro program (Geneva, Switzerland) [32] and were classified according to WHO growth standards using the corresponding Z-score cut-off points for length for age and weight for length to be classified as Adequate, Low length risk, Low length or Severely low length and as Obesity, Overweight Risk of Overweight, Adequate and Risk of Acute Malnutrition [33]. Additionally, undernutrition in children under six months was determined using a MUAC cut-off point of <11 cm. For children aged 6–24 months, severe and moderate acute undernutrition was determined according to the MUAC cut-off points of <11.5 cm and 11.5 to 12.5 cm, respectively [34]. The nutritional status of pregnant women was classified according to the Body Mass Index (BMI) WHO classification, calculated as pre-pregnancy weight in kilograms divided by height in m^2^: Underweight BMI < 18.5 kg/m^2^, Adequate weight BMI ≥ 18.5 kg/m^2^ & BMI ≤ 24.99 kg/m^2^, Overweight BMI ≥ 25 kg/m^2^ & BMI ≤ 29.99 kg/m^2^ and Obesity BMI ≥ 30 kg/m^2^. The MUAC cut-off point < 24 cm [35] was used to identify underweight women. We compared different variables of interest before and after the emergency using McNemar’s test for paired dichotomous variables and the McNemar–Bowker test for paired variables with more than two categories. To compare paired continuous variables before and after the emergency, the Wilcoxon signed-rank test was applied. All analyses were conducted using STATA version 14.0 (StataCorp LLC, College Station, TX, USA). A *p* value of less than 0.05 was considered statistically significant.

### 2.4. Ethical Considerations

Prior to conducting the study, approval was obtained from the Research Committee and the Ethics Committee (CI: 1953) of the NIPH. All participants gave their oral and written consent to participate in the study. It is worth mentioning that, in the post-emergency context, and to ensure the participation of the study population, an economic incentive ($10 USD) was provided to accelerate recruitment and maximize participant reach within each selected healthcare unit.

## 3. Results

This study involved 20 primary healthcare units. Data were collected from 239 children and their mothers or caregivers, and 76 pregnant women.

Among the sociodemographic characteristics, most of the mothers or primary caregivers were from the Acapulco region (74.6%, *n* = 235), with a mean age of 27.7 (±7.7 SD). Most participants had secondary education (38.7%, *n* = 122), were living with a partner (either cohabiting or married) (92.4%, *n* = 291) and had a low SES (67.6%, *n* = 213) (Table 1).

Following the emergency, a decline in both access to and quality of water for drinking and domestic use was identified across the study population (Table 2). The proportion of children and pregnant women with access to safe drinking water decreased significantly from 74.6% before the emergency to 29.3% afterward. Similarly, the use of water for basic needs declined after the emergency: drinking (94.9% vs. 60.3%) cooking (97.1% vs. 61%), personal hygiene (93% vs. 53.7%), and other domestic purposes (89.8% vs. 44.8%) (*p* < 0.05). Likewise, a decrease in the prevalence of “good water quality” was recorded after the emergency (76.2% vs. 33.3%), with a notable increase in “poor water quality” (1.0% vs. 24.1%), (*p* < 0.001). These trends were consistent across all study groups (Appendix A Table A1).

Regarding the food items available in the food pantries delivered to families (*n* = 25 for caregivers of children aged 0–6 months and *n* = 69 for caregivers of children aged 6–24 months), caregivers of children aged 6–24 months most commonly reported receiving tuna or sardines, beans, and rice. In contrast, caregivers of children under 6 months primarily reported receiving cooking oil and rice. With respect to breast milk substitutes (BMS), 48% (*n* = 12) of caregivers of children aged 0–6 months and 58% (*n* = 40) of those of children aged 6–24 months reported receiving powdered milk. In addition, caregivers of children aged 0–6 months reported that they did not receive CIF. In contrast, among caregivers of children aged 6–24 months, 9% (*n* = 6) received CIF and 7% (*n* = 5) received processed baby food. Only 3% (*n* = 2) reported receiving sugar-sweetened beverages.

**Figure 2:**
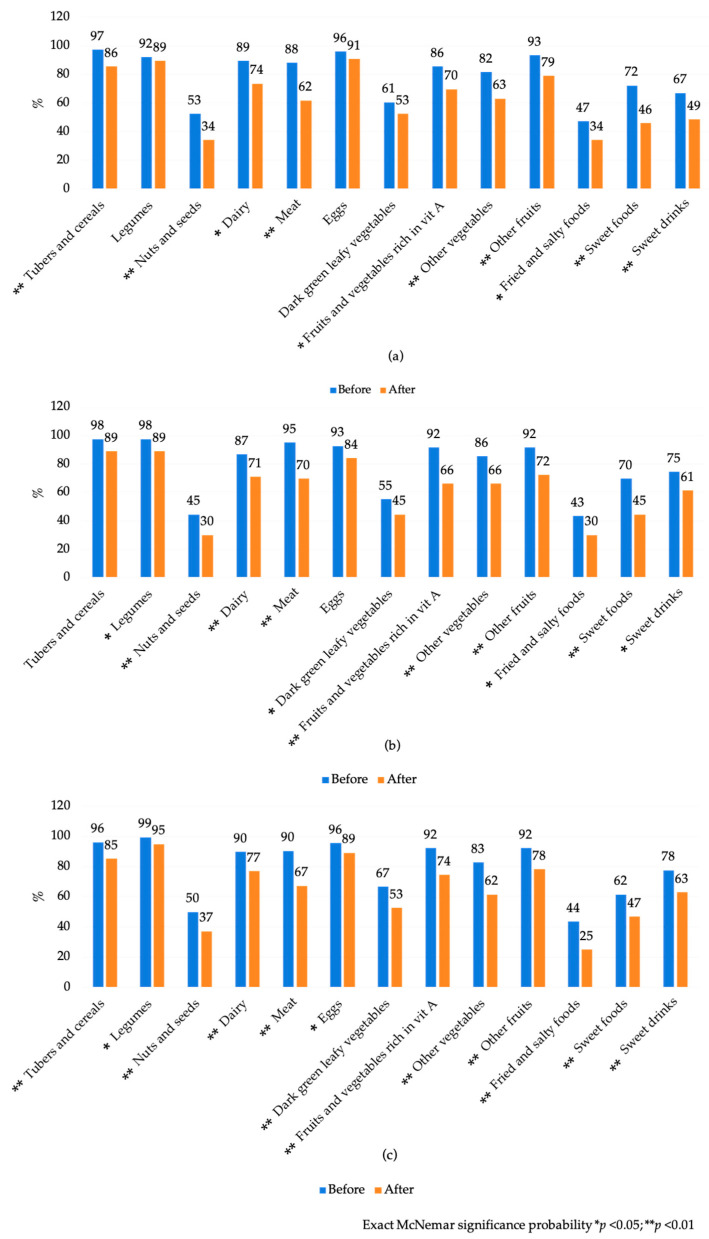
Food groups consumption among pregnant and breastfeeding women group before and after Hurricane John. Guerrero, México. 2024. (**a**) Consumption among pregnant women; (**b**) consumption among caregivers of children aged 0–6 months; and (**c**) consumption among caregivers of children aged 6–24 months before and after Hurricane John.

In terms of maternal food group consumption, a statistically significant decrease was observed in the consumption of most of the groups evaluated (*p* < 0.05). Exceptions were found for legumes, eggs, and dark green leafy vegetables among pregnant women, and cereals and tubers as well as eggs among caregivers of children aged 0–6 months (*p* > 0.05). Among caregivers of children aged 6–24 months, a decrease in consumption was reported for all food groups evaluated before and after the emergency (*p* < 0.05). (Figure 2 and Appendix A Table A2) Furthermore, there was a 37% increase in the prevalence of women consuming only two meals per day, while the proportion of women who ate three meals a day decreased to 35.1% (Appendix A Table A3).

### 3.1. Infant and Young Child Feeding Practices

**Figure 3:**
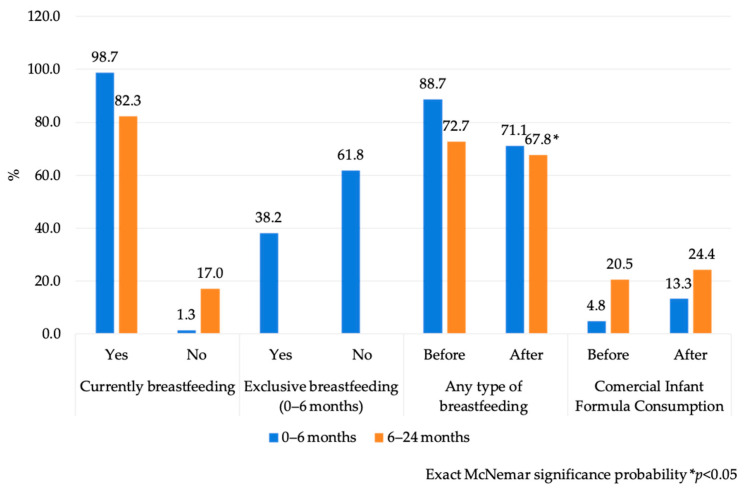
Breastfeeding practices before and after Hurricane John, reported by mothers or principal caregivers of the infants. Guerrero, México. 2024.

At the time of the interview, most women were breastfeeding: 98.7% (*n* = 76) of those with infants under 6 months and 82.3% (*n* = 121) of those with children 6–24 months. The prevalence of exclusive breastfeeding was 38.2% (*n* = 29). Self-reported breastfeeding practices decreased significantly following the emergency, particularly among infants aged 0–6 months (88.7% before vs. 71.1% after; *p* < 0.05). In contrast, breastfeeding rates among children 6–24 months remained relatively stable after the emergency (72.7% before vs. 67.8% after; *p* > 0.05). An increase in the consumption of CIF was also observed after the emergency in both age groups (from 4.8% to 13.3% in the 0–6-month age group, *p* = 0.016 and from 20.5% to 24.4% in the 6–24-month age group, *p* > 0.05) (Figure 3). The main reasons mentioned for initiating CIF feeding were insufficient breastmilk (28.4% for infants <6 months; 26.8% for children 6–24 months), maternal illness (17.0% and 8.9%, respectively), infant refusal of breastmilk (7.6% and 9.0%, respectively), perceived infant hunger (5.7% only for infants <6 months) and maternal choice (9.4% and 8.9%, respectively).

Regarding children aged 6–24 months, a statistically significant increase was observed in the mean number of food groups consumed before and after the emergency (3.6 ± 2.1 vs. 4.5 ± 1.5; *p* < 0.05). Similarly, the proportion of infants and young children who achieved minimum dietary diversity increased from 57.0% (*n* = 89) before the hurricane to 75.6% (*n* = 118) afterward (*p* < 0.05). When analyzing those who received food supplies after the hurricane, it was found that 42% of those who increased their dietary diversity experienced this benefit.

### 3.2. Maternal and Infant Nutritional Status

**Figure 4:**
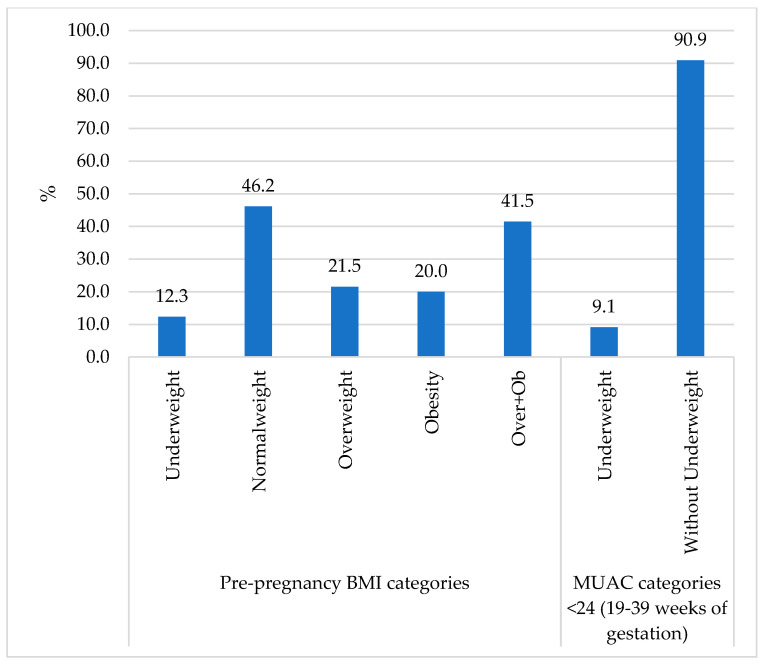
Nutritional status in pregnant women after Hurricane John. Guerrero, México. 2024.

Regarding nutritional status, pregnant women had an average weight of 67.3 kg, height of 1.55 m, a BMI of 28.1 kg/m^2^ and a MUAC of 29.2 cm. Based on pre-pregnancy BMI, 12.3% (*n* = 8) were underweight, with 20.6% (*n* = 7) of these women aged 16 to 24 years. Additionally, 21.5% (*n* = 14) of pregnant women were overweight, of whom 57% (*n* = 8) were between 16 and 29 years old. Obesity was observed in 20% (*n* = 13) of pregnant women, of which 46% (*n* = 6) were between 25 and 29 years old. Based on the MUAC measurement, only 9.1% (*n* = 5) of pregnant women were underweight. (Figure 4).

Infants under 6 months had a mean age of 2.6 ± 1.5 months; 7.2% were stunted, 9.6% were classified as having risk of acute malnutrition or moderate acute malnutrition (MAM), and one case of severe acute malnutrition (SAM) was identified. Children aged 6–24 months had a median age of 13.1 ± 4.6 months; 13.9% were stunted, and 14.8% were classified as having risk of acute malnutrition or MAM. Based on MUAC measurements, 8.4% of children from both age groups were classified as malnourished. Detailed anthropometric indicators by age group are presented in Table 3.

## 4. Discussion

This study presents cross-sectional data collected before and after the emergency, reported by participants from two provinces in Guerrero, Mexico, both of which were severely affected by Hurricane John in September 2024.

While the preparedness and response efforts of Mexican authorities to recent emergencies have followed national and state civil protection guidelines [36], a substantial gap persists in ensuring that these actions adequately meet the distinct nutritional needs of pregnant and lactating women, infants, and young children. Moreover, current response strategies remain only partially aligned with national and international recommendations on nutrition in emergency contexts.

Our findings indicate that the disaster negatively affected several aspects of maternal and IYCF practices. These results are consistent with previous studies, particularly regarding the decline in breastfeeding, which has been associated with increased morbidity and mortality in emergency contexts due to the higher use of CIF (an increase in our study of 8.5 and 3.9 percentage points for infants 0–6 and 6–24 months, respectively). The latter increases the risk of illness and death resulting from contamination by unsafe water, inadequate sterilization, and early cessation of breastfeeding [18]. IYCF practices before the emergency play a crucial role in shaping the required emergency response [3].

The prevalence of exclusive breastfeeding among infants under 6 months in this study (38%) was similar than the national average reported by the ENSANUT-Continua 2021–2024, which stands at 34.1% (Data not publicized). In a country like Mexico, where only one-third of infants are exclusively breastfed during the first six months, and mixed feeding practices are common, emergency nutrition responses are particularly complex and require tailored planning that accounts for this diversity. Governments should recognize that high breastfeeding rates enhance community resilience and consider investment in breastfeeding support as a key component of emergency preparedness [11].

Regarding complementary feeding practices, contrary to expectations, an increase in the consumption of food groups was observed after the emergency. The prevalence of minimum dietary diversity before the emergency was lower than the national average (57.0% in our study vs. 67.0% according to ENSANUT 2022 [28] and increased significantly afterward, reaching 75.6% of infants and young children who received a diverse diet. A possible explanation for these results is that children were older at the time of the post-emergency assessment and had started consuming a wider variety of foods. Evidence suggests that as children grow, the number and variety of food groups consumed should naturally increase, reflecting normal dietary progression during infancy and early childhood [29].

In emergency contexts, the distribution of CIF tends to increase its availability and discourage breastfeeding [11]. The most common violations of the Code involve the acceptance of CIF donations and their untargeted distribution [13]. In many cases, health and nutrition responders either lacked awareness of, or did not adhere to, established protocols for infant feeding in emergencies. The Infant and Young Child Feeding in Emergencies (IYCF-E) framework, promoted by the Infant Feeding in Emergencies (IFE) Core Group, underscores the importance of preparedness, integration of infant feeding into emergency policies, and capacity building across all response levels. It further calls for context-specific actions to protect recommended feeding practices, ensure the safe use of CIF only under professional supervision, and address the nutritional needs of pregnant and lactating women through coordinated, multisectoral efforts.

Our results show violations of the Code, including the indiscriminate donation of CIF and other breast-milk substitutes (BMS) among the study population. Among families who received food pantries, nearly half of the caregivers of children aged 0–6 months (48%) and more than half of those of children aged 6–24 months (58%) received powdered milk as part of these distributions. In the 6–24-month group, almost 9% received CIF, 7% received processed baby food, and 3% received sugar-sweetened beverages as part of the food aid packages. This factor may also contribute to early cessation of breastfeeding.

Our findings also revealed that maternal beliefs contributed to the early introduction of CIF, as reflected in the increased proportion of children consuming CIF after the emergency. Some mothers reported concerns such as insufficient breast milk production, perceptions that their baby remained hungry, or that the infant no longer wanted to breastfeed.

In Mexico, a variety of cultural beliefs influence breastfeeding practices. For instance, emotional experiences such as fright (*susto*) or intense anger (*coraje*) during pregnancy or lactation are believed to negatively affect breast milk production (“cutting off” the milk) and to cause illness, malnutrition, or weakness in children. These events are also associated with folk illnesses such as *empacho*, a digestive discomfort. It is further believed that exposure to sun or fire, or returning home tired or agitated, can spoil breast milk and harm the baby [17,37,38].

These culturally rooted beliefs may explain why some women report having “no milk” during emergencies. Although there is no physiological basis for breast milk cessation due to *susto*, acute stress can inhibit oxytocin release and temporarily disrupt milk ejection. Such disruptions, documented in emergency contexts, highlight the need to reassure mothers that negative emotions do not eliminate milk production [39]. Conversely, breastfeeding itself can reduce stress through oxytocin release and skin-to-skin contact, acting as a protective mechanism during emergencies. Similar beliefs have been documented in other contexts. For example, in Haiti, it is commonly believed that strong emotions or bad news can stop breast milk production, and that exposure to solar heat can warm the breast milk and make the child ill [40].

It is essential in emergency situations to ensure that pregnant and breastfeeding women receive adequate information about the importance of continuing breastfeeding. It is equally important to provide guidance to other primary caregivers about safe and culturally acceptable alternatives when breastfeeding is not feasible. In such cases, the promotion and availability of wet nursing or donor human milk in shelters become particularly relevant [18]. This aligns with WHO recommendations emphasizing comprehensive approaches to protect, promote, and support breastfeeding during emergencies. For instance, during the COVID-19 pandemic, WHO recommended that health workers explore the feasibility of relactation, wet nursing, or donor human milk when mothers are too ill to breastfeed or express milk, considering cultural context, maternal acceptability, and available services [41].

When breastfeeding is not possible, it is critical to ensure that all supplies necessary for the safe preparation of CIF are available. CIF should only be distributed to mothers and caregivers by trained health professionals, and only after an individual assessment confirms that no other feeding options are viable, such as expressed breast milk, donor human milk, or relactation. This applies to specific cases such as severe maternal illness, temporary separation, HIV-related replacement feeding, or maternal absence or death [3,42].

Access to safe water, sanitation, and hygiene (WASH) was negatively affected after the emergency, increasing the population’s vulnerability to infections and malnutrition. In line with this, the study also documented cases of moderate acute malnutrition (MAM) among children under 24 months in the assessed localities. Without appropriate nutritional support, these children are at risk of progressing to SAM, significantly increasing their mortality risk. The inclusion of ready-to-use therapeutic foods (RUTF) should therefore be considered as part of a targeted strategy to improve their nutritional status, alongside efforts to ensure access to safe water and healthy, age-appropriate foods.

The consequences of climate change, including droughts, fires, extreme heat, rising sea levels, and catastrophic storms, are expected to intensify [20]. Moreover, Mexico’s high vulnerability to hurricanes and earthquakes highlights the need to strengthen the capacity and skills of health workers and communities to respond effectively to emergencies affecting mothers and children.

The study has certain limitations. Although random sampling of healthcare units affected by the hurricane would have been ideal, fieldwork was limited by contextual conditions beyond the research team’s control (e.g., insecurity and violence). On the other hand, the limitations related to the small, opportunistic sample and the retrospectively data collection during a specific period should be considered when interpreting and generalizing the findings. Despite these constraints, the study provides valuable information on maternal and child nutrition practices in a real emergency context, an area where evidence remains scarce or is limited to literature review. While it would have been ideal to have baseline data prior to the disaster, the unpredictable nature of emergencies makes this difficult. The comparison of retrospective before and after emergency practices reported by the same participants may also be subject to recall bias. However, this bias is likely non-differential, given the consistent administration of the instrument by trained personnel and the use of a questionnaire designed to minimize recall errors. In addition, although retrospective information may have reduced precision, the relatively short recall period, and the standardized application of instruments may have mitigated this concern and did not compromise the internal validity of the study.

These findings highlight the importance of continued data collection and documentation to inform future research and strengthen maternal and IYCF practices in emergency preparedness and response. A key strength of this study is its contribution to an understudied area: the influence of natural disasters on maternal and child feeding and nutrition in Latin America. Few studies have compared population-level nutrition practices before and after disaster events in the region.

## 5. Lessons Learned and Key Recommendations

The findings from this study underscore the urgent need to strengthen preparedness and response systems for maternal and child nutrition in emergencies. After Hurricane John, essential services such as access to safe water and sanitation were severely disrupted, compromising hygiene and increasing risks related to infant feeding. Breastfeeding practices declined while the use of CIF rose, highlighting the need for culturally sensitive communication and sustained breastfeeding support during crises.

In addition, maternal consumption of different food groups decreased after the emergency, reflecting deteriorating food security and access to nutritious foods. Cases of moderate acute malnutrition were identified among children under 24 months, emphasizing the need to integrate specialized nutrition interventions, such as ready-to-use therapeutic foods (RUTF), into emergency response plans to prevent severe outcomes and protect the most vulnerable.

To strengthen preparedness and improve the response to natural disasters or other types of emergencies, particularly those affecting mothers and young children, we propose the following set of actionable recommendations for key actors involved in emergency management. These recommendations have been based on our study and on international guidance including the Academy of Breastfeeding Medicine Position Statement on Breastfeeding in Emergencies and other emergency guidelines (Table 4).

The underlying causes and factors that determine disaster risk must be addressed so that institutional efforts are not limited to emergency response or to promoting fragmented reconstruction actions that do not contribute to reducing vulnerability [25].

The impact of Hurricanes Otis (2023) and John (2024) in the state of Guerrero provided an opportunity to design, based on a real-life example, specific guidance to inform nutrition responses in future emergencies, and to establish a Maternal and Child Nutrition Technical Working Group for emergencies, bringing together key institutions, groups, and sectors involved in maternal and child nutrition.

## 6. Conclusions

In conclusion, the findings of this study support existing evidence that indicates the urgent need for a comprehensive action plan to respond to climate-related and other emergencies. Such a plan must prioritize rapid and targeted care for vulnerable populations, including pregnant and breastfeeding women, as well as children under 2 years of age, who are often adversely impacted by disasters. This study provides valuable insights into the persistent challenges facing IYC-E. In contexts like Mexico, emergency responses must be comprehensive, culturally sensitive, and adapted to the heterogeneity of pre-existing IYCF practices. Maternal and child nutrition should be recognized as a central component of all emergency interventions to help mitigate the negative effects of disasters on their health and nutrition status. Strengthening preparedness and response capacity at the municipal, state and national levels in Mexico is essential. This includes integrating maternal and IYCF practices into emergency planning, ensuring coordinated action across sectors, and investing in systems that protect and promote optimal nutrition practices during crises.

## Figures and Tables

**Table 1:** General characteristics of the study population by study group. Guerrero, México. 2024.

	Pregnant Women (*n* = 76)		Caregivers from Infants < 6 m (*n* = 83)		Caregivers from Children 6–24 m (*n* = 156)		Total (*n* = 315)	
	*n*	%	*n*	%	*n*	%	*n*	%
Health Jurisdiction								
Acapulco	59	77.6	62	74.7	114	73.1	235	74.6
Costa Grande	17	22.4	21	25.3	42	26.9	80	25.4
Age (mean ± SD)	25.9	6.3	26.2	6.2	29.3	8.7	27.7	7.7
Categorical age								
16–24	36	47.4	35	42.2	50	32.0	121	38.4
25–29	23	30.3	24	28.9	38	24.4	85	27.0
30–39	14	18.4	21	25.3	54	34.6	89	28.2
>40	3	4.0	3	3.6	14	9.0	20	6.3
Marital status								
Free union (Cohabiting)	38	50.0	48	57.8	68	43.6	154	48.9
Separated/divorced	1	1.3	0	0.0	6	3.8	7	2.2
Married	34	44.7	26	31.3	77	49.4	137	43.5
Single woman	3	4.0	8	9.6	5	3.2	16	5.1
Widow	0	0.0	1	1.2	0	0.0	0.32	0.3
Education								
None	1	1.3	1	1.2	1	0.6	3	0.9
Primary School	11	14.5	7	8.4	19	12.2	37	11.7
Secondary School	33	43.4	28	33.7	61	39.1	122	38.7
High School	29	38.2	37	44.6	54	34.6	120	38.1
Technical or commercial	0	0.0	0	0.0	3	3.0	3	0.9
Bachelor’s Degree	2	2.6	10	12.0	18	11.5	30	9.5
Socioeconomic Level								
High	0	0.0	0	0.0	1	0.6	1	0.3
Medium	14	18.4	12	14.5	37	23.7	63	20
Low	53	69.7	66	79.5	94	60.3	213	67.6
Very low	9	11.8	5	6.0	24	15.4	38	12.1

SD: Standard deviation.

**Table 2:** Access to water and sanitation before and after the emergency by Hurricane John. Guerrero, México. 2024.

	Total (*n* = 315)				
	Before		After		*p* Value *
	*n*	%	*n*	%	
Access to drinking water					
Yes	235	74.6	92	29.3	0.000
No	80	25.4	222	70.7	
Water to meet needs such as:					
Drinking	299	94.9	190	60.3	0.000
Cooking	306	97.1	192	61	0.000
Personal hygiene (washing or bathing)	293	93	169	53.7	0.000
Other household purposes (cleaning the house, floor, etc.)	283	89.8	141	44.8	0.000
There is not enough water to meet any of the above needs	6	1.9	98	31.1	0.000
Water quality					
Good (no smell, color or taste)	240	76.2	105	33.3	** 0.001
Regular (the water is cloudy)	71	22.5	133	42.2	
Poor (has smell, color or taste)	3	1	76	24.1	
Diseases associated with water consumption					
Diarrheal diseases (Yes)	31	9.8	43	13.7	** 0.01
Diseases such as dysentery, typhoid fever, cholera, hepatitis (Yes)	3	1	5	1.6	
Skin infections (Yes)	4	1.3	21	6.7	

* McNemar Test; ** McNemar–Bowker Test.

**Table 3:** Nutritional status of children < 24 months after Hurricane John. Guerrero, Mexico. 2024.

	<6 m (*n* = 83)		6–24 m (*n* = 156) *	
Indicators				
L/A Categories	n	%	n	%
Adequate	67	80.7	89	58.6
Low length risk	10	12.0	42	27.6
Low length	4	4.8	15	9.9
Severely low length	2	2.4	6	4.0
W/L Categories	n	%	n	%
Obesity	-	-	2	1.3
Overweight	5	6.0	11	7.1
Risk of Overweight	17	20.5	19	12.2
Adequate	52	62.7	101	64.7
Risk of Acute Malnutrition	5	6.0	19	12.2
MAM	3	3.6	4	2.6
SAM	1	1.2	0	0.0
MUAC Categories	n	%	n	%
Normal	76	91.6	143	91.7
With malnutrition	7	8.4	-	-
Risk of Acute Malnutrition	-	-	11	7.1
MAM	-	-	2	1.3

BMI: Body Mass Index; MAM: Moderate Acute Malnutrition; MUAC: Mid Upper Arm Circumference; SAM: Severe Acute Malnutrition; SD: Standard Deviation; * L/A greater than −6 SD were removed.

**Table 4:** Actionable recommendations for key actors involved in emergency management.

Key Actors	Recommendations
Federal Government and Decision-Makers	Recognize breastfeeding and complementary feeding in emergencies (IYCF-E) as national priorities, integrating them into national emergency, nutrition, and food security policies. Allocate dedicated budget lines for IYCF-E.
Ministry of Health	Establish intersectoral IYCF-E working groups at national and state levels (including health, education, civil protection, social development, and WASH). Pre-position essential supplies, MMS, MNP, and RUTF, to ensure rapid deployment. Ensure emergency plans include skilled breastfeeding counselors and trained personnel to manage maternal and infant feeding needs.
Civil Protection, Red Cross, and Emergency Response Agencies	Identify and register pregnant women, infants, and caregivers early during emergencies, ensuring that mothers and infants remain together in shelters. Designate safe, private spaces for breastfeeding and milk expression in shelters. Monitor and strictly regulate the donation and distribution of CIF, authorizing it only after individual assessment.
Army and Food Distribution Agencies (e.g., DIF)	Train staff on the importance of maternal and child nutrition in emergencies. Ensure that distributed foods are healthy, minimally processed, and appropriate for young children, excluding ultra-processed foods and sugar-sweetened beverages. Prioritize pregnant and lactating women and young children in food and water provision. Equip shelters with basic utensils and supplies to facilitate appropriate complementary feeding.
Primary Healthcare Units	Maintain updated community registries of pregnant women, children under two, and their caregivers to enable targeted response. Promote breastfeeding as part of emergency preparedness and address harmful cultural beliefs (e.g., that emotional distress stops milk production). Train staff to identify and manage malnutrition and to provide micronutrient supplementation during and after emergencies. Include the assessment of maternal mental health in emergency settings, given the substantial emotional burden such situations can impose.
Local Governments and Municipalities	Guide the public on appropriate donations (e.g., food, medicines, utensils) Monitor expiry dates and safety of donated supplies.
All Levels of Government	Regulate the marketing and donation of commercial infant foods and BMS, ensuring compliance with the Code. Avoid indiscriminate distribution of BMS and discourage items that violate the Code.

IYCF-E: Infant and Young Child Feeding in Emergencies; MMS: Multiple Micronutrient Supplements; MNP: Micronutrient Powders; CIF: Commercial Infant Formula; WASH: Water, Sanitation, and Hygiene; RUTF: Ready-to-Use Therapeutic Foods.

## Data Availability

Requests to access the datasets should be directed to the corresponding author, AB.

## Abbreviations

The following abbreviations are used in this manuscript:

AMAI	Mexican Association of Market Research Agencies
BMI	Body Mass Index
BMS	Breastmilk Substitute
CDC	Center for Disease Control
CIF	Commercial Infant Formula
ENSANUT	National Health and Nutrition Survey
IFE	Infant Feeding in Emergencies
IYCF-E	Infant and Young Child Feeding in Emergencies
IYCF	Infant and Young Child Feeding
MAM	Moderate Acute Malnutrition
MMSs	Multiple Micronutrient Supplements
MNPs	Micronutrient Powders
MUAC	Mid-Upper Arm Circumference
NIPH	National Institute of Public Health of Mexico
OG-IFE	Operational Guidance on Infant and Young Child Feeding in Emergencies
REDCap RUFT	Research Electronic Data Capture Ready-to-use therapeutic foods
SAM	Severe Acute Malnutrition
SD	Standard Deviation
SES	Socioeconomic Status
SSGro	Secretaría de Salud de Guerrero (by its acronym in Spanish)
UNICEF	United Nations International Children’s Emergency Fund
WASH	Water, Sanitation and Hygiene
WHO	World Health Organization

## Appendix A

**Table A1:** Access to water and sanitation before and after the emergency by study group. Guerrero, México. 2024.

	Pregnant Women (*n* = 76)					Caregivers from Infants < 6 m (*n* = 83)					Caregivers from Children 6–24 m (*n* = 156)				
	Before		After		*p* Value *	Before		After		*p* Value *	Before		After		*p* Value *
Access to drinking water	n	%	n	%		n	%	n	%		n	%	n	%	
Yes	60	79.0	22	29.0	0.000	58	69.9	27	32.5	0.000	117	75.0	43	27.6	0.000
No	16	21.1	53	69.7		25	30.1	56	67.5		39	25.0	113	72.4	
Water to meet needs such as:															
Drinking	72	94.7	40	52.6	0.000	79	96.3	48	58.5	0.000	148	94.3	102	65.0	0.000
Cooking	74	97.4	39	51.3	0.000	80	97.6	48	58.5	0.000	152	96.8	105	66.9	0.000
Personal hygiene (washing or bathing)	75	98.7	42	55.3	0.000	75	91.5	42	51.2	0.000	143	91.1	85	54.1	0.000
Other household purposes	75	98.7	33	43.4	0.000	70	85.4	27	32.9	0.000	138	87.9	81	51.6	0.000
There is not enough water to meet any of the above needs	2	2.6	30	39.5	0.000	1	1.2	25	30.5	0.000	3	1.9	43	27.4	0.000
Water quality															
Good (no smell, color or taste)	63	82.9	21	27.6	** 0.001	61	73.2	32	38.6	** 0.001	116	74.8	52	33.3	** 0.001
Regular (the water is cloudy)	13	17.1	30	39.5		22	26.8	33	39.8		36	23.2	70	45.5	
Poor (has smell, color or taste)	0	0.0	25	32.9		0	0.0	18	21.7		3	1.9	33	21.2	
Diseases associated with water consumption															
Diarrheal diseases (Yes)	8	10.5	9	11.8	** 0.32	6	7.3	6	7.3	** 0.99	17	10.8	28	17.8	** 0.012
Diseases such as dysentery, typhoid fever, cholera, hepatitis (Yes)	0	0.0	2	2.6		2	2.4	2	2.4		1	0.6	1	0.6	
Skin infections (Yes)	1	1.3	7	9.2		2	2.4	4	4.9		1	0.6	10	6.4	

* McNemar Test; ** McNemar-Bowker Test.

**Table A2:** Food group consumption among pregnant women and caregivers of children under 24 months before and after Hurricane John, Guerrero, Mexico, 2024.

	Pregnant Women (*n* = 76)						Caregivers 0–6 Months (*n* = 83)				Caregivers 6–24 Months (*n* = 156)				
	Before		After			Before		After			Before		After		
Group	*n*	%	*n*	%	*p* Value *	*n*	%	n	%	*p* Value *	*n*	%	*n*	%	*p* Value *
Tubers and cereals	74	97.4	65	85.5	0.004	81	97.6	74	89.2	0.065	150	96.2	133	85.3	0.001
Legumes	70	92.1	68	89.5	0.727	81	97.6	74	89.2	0.039	155	99.4	148	94.9	0.039
Nuts and seeds	40	52.6	26	34.2	0.003	37	44.6	25	30.1	0.004	78	50.0	58	37.2	0.003
Dairy	68	89.5	56	73.7	0.023	72	86.8	59	71.1	0.002	140	89.7	120	76.9	0.001
Meat	67	88.2	47	61.8	0.000	79	95.2	58	69.9	0.000	141	90.4	105	67.3	0.000
Eggs	73	96.1	69	90.8	0.289	77	92.8	70	84.3	0.092	149	95.5	139	89.1	0.031
Dark green leafy vegetables	46	60.5	40	52.6	0.210	46	55.4	37	44.6	0.035	104	66.7	82	52.6	0.001
Fruits and vegetables rich in vit A	65	85.5	53	69.7	0.012	76	91.6	55	66.3	0.000	144	92.3	116	74.4	0.000
Other vegetables	62	81.6	48	63.2	0.001	71	85.5	55	66.3	0.001	129	82.7	96	61.5	0.000
Other fruits	71	93.4	60	79.0	0.001	76	91.6	60	72.3	0.000	144	92.3	122	78.2	0.000
Fried and salty foods	36	47.4	26	34.2	0.031	36	43.4	25	30.1	0.027	68	43.6	39	25.0	0.000
Sweet foods	55	72.4	35	46.1	0.000	58	69.9	37	44.6	0.000	96	61.5	73	46.8	0.000
Sweet drinks	51	67.1	37	48.7	0.004	62	74.7	51	61.5	0.043	121	77.6	98	62.8	0.000

* Exact McNemar significance probability.

**Table A3:** Number of meals consumed per day before and during Hurricane John, by study group. Guerrero, Mexico, 2024.

		Before		After		
Group	Meals/Day	*n*	%	*n*	%	Bowker χ^2^ (*p* Value)
Pregnant women (*n* = 76)	1	2	2.63	5	6.6	26.3 (*p* = 0.003)
	2	18	23.7	39	51.3	
	3	49	64.5	30	39.5	
	4	6	7.9	2	2.6	
	5	1	1.3	0	0.0	
Caregivers of children < 6 months (*n* = 83)	1	1	1.2	6	7.3	43.0 (*p* < 0.001)
	2	18	21.7	49	59.8	
	3	54	65.1	27	32.9	
	4	8	9.6	0	0.0	
	5	2	2.4	0	0.0	
Caregivers of children 6–24 months (*n* = 156)	1	0	0.0	15	9.9	68.3 (*p* < 0.001)
	2	36	23.1	95	62.5	
	3	105	67.3	39	25.7	
	4	15	9.6	3	2.0	
All groups	1	3	1.0	26	8.4	150.7 (*p* < 0.001)
	2	72	22.9	183	59.0	
	3	208	66.0	96	31.0	
	4	29	9.2	5	1.6	
	5	3	1.0	0	0.0	
